# A psychology of the human brain–gut–microbiome axis

**DOI:** 10.1111/spc3.12309

**Published:** 2017-04-18

**Authors:** Andrew P. Allen, Timothy G. Dinan, Gerard Clarke, John F. Cryan

**Affiliations:** ^1^ Dept Psychiatry & Behavioural Neuroscience/APC Microbiome Institute University College Cork Cork Ireland; ^2^ Dept Anatomy & Neuroscience/APC Microbiome Institute University College Cork Cork Ireland

**Keywords:** Microbiome, brain‐gut‐microbiome axis, stress, cognition, mood

## Abstract

In recent years, we have seen increasing research within neuroscience and biopsychology on the interactions between the brain, the gastrointestinal tract, the bacteria within the gastrointestinal tract, and the bidirectional relationship between these systems: the brain–gut–microbiome axis. Although research has demonstrated that the gut microbiota can impact upon cognition and a variety of stress‐related behaviours, including those relevant to anxiety and depression, we still do not know how this occurs. A deeper understanding of how psychological development as well as social and cultural factors impact upon the brain–gut–microbiome axis will contextualise the role of the axis in humans and inform psychological interventions that improve health within the brain–gut–microbiome axis. Interventions ostensibly aimed at ameliorating disorders in one part of the brain–gut–microbiome axis (e.g., psychotherapy for depression) may nonetheless impact upon other parts of the axis (e.g., microbiome composition and function), and functional gastrointestinal disorders such as irritable bowel syndrome represent a disorder of the axis, rather than an isolated problem either of psychology or of gastrointestinal function. The discipline of psychology needs to be cognisant of these interactions and can help to inform the future research agenda in this emerging field of research. In this review, we outline the role psychology has to play in understanding the brain–gut–microbiome axis, with a focus on human psychology and the use of research in laboratory animals to model human psychology.

## 1 INTRODUCTION

Most people today think they belong to a species that can be master of its destiny. This is faith, not science. We do not speak of a time when whales or gorillas will be masters of their destinies. Why then humans?

John N. Gray, (2002), *Straw Dogs: Thoughts on Humans and Other Animals*

We are living in a microbial world, having coevolved with the microbiota—the trillions of microorganisms (bacteria, viruses, archae, fungi) that inhabit our bodies and in particular our intestines (McFall‐Ngai et al., 2013; Theis et al., 2016). Over recent years, the microbiota has become a topic of increasing research interest within biology, and the brain–gut–microbiome axis has become a major research area at the interface of neuroscience and microbiology (e.g., Cryan & Dinan, 2012). The study of how microbes within the body can interact with human brain and behaviour can offer a more complete understanding of human psychology.

In attempting to build a scientific psychology, Freud and others within the psychoanalytical school of thought placed a great emphasis on unconscious processes in human psychology (e.g., Freud, 1895, also cf. Sulloway, 1979, for an interesting discussion). The psychoanalytic emphasis on the unconscious has been met with attempts at integration with contemporary cognitive views of the unconscious (e.g., Epstein, 1994); the brain–gut–microbiota axis represents a further, physiological means through which unconscious processes may impact on our behaviour. If some information has relevance to a person's goals, it is not simply processed at a formal, logical level (sometimes referred to as *Type 2* processes in thinking; e.g., Evans, 2007) but also leads to a subjective appraisal that is driven by an emotional response that is in turn tied up with a bodily response to the information. This emotional response will interact with the gut as well as, for example, cardiovascular, respiratory, and hormonal systems. However, until relatively recently, there has been little research integrating the study of the mind and brain with the microbiological study of the bacteria in the human body, especially the gut. The surge of interest in the brain–gut–microbiome axis has been so great that Mayer, Knight, Mazmanian, Cryan, and Tillisch (2014) have described it as nothing less than a paradigm shift in neuroscience; this is clearly an area in which the discipline of psychology could make a substantial contribution.

In this review, we outline the brain–gut–microbiome axis and its relevance to numerous aspects of psychology. We discuss changes in the gut microbiome across development and its relevance for psychological development. We also place the brain–gut–microbiome axis within a broader social and cultural milieu, highlighting the potential role of this axis in interpersonal social psychology, as well as how culture can moderate factors such as treatment of brain–gut–microbiome axis disorder. Irritable bowel syndrome is discussed as a condition of particular interest, and we discuss more broadly the potential for interventions to improve psychological health and ameliorate disorders of the brain–gut–microbiome axis. We raise some factors for psychologists in clinical practice to consider, and, given the novelty of this field, we suggest potential future directions for research in this area. Finally, we discuss the potential for an increased emphasis on the brain–gut–microbiome axis to disrupt a number of biases within psychology as a discipline.

## 2 THE GUT MICROBIOME, BRAIN, AND BEHAVIOUR

The gastrointestinal system contains a vast array of microorganisms, and the intestines in particular are home to a rich diversity of bacteria. These bacteria serve a wide range of physiological functions, including digestion and preservation of the integrity of the intestinal barrier (Grenham, Clarke, Cryan, & Dinan, 2011). The gastrointestinal tract also contains an enteric nervous system comprising an interconnected network of neurons, similar in number to the neurons in the spinal cord (Sasselli, Pachnis, & Burns, 2012). The brain sends signals to the gut, which impacts upon its sensory and secretory function, and in return receives visceral information from the gut (Grenham et al., 2011; Montiel‐Castro, González‐Cervantes, Bravo‐Ruiseco, & Pacheco‐López, 2013). A great volume of interoceptive information is sent from the gut to the brain, and much of this will not be processed consciously (Mayer, 2011), joining a broader system of interoceptive information coming from the body as a whole (Craig, 2003). By relaying this information to the central nervous system, the gut can thus play a key role in generating somatic markers (cf. Damasio, Everitt, & Bishop, 1996; Reimann & Bechara, 2010). Bidirectional communication between the gut microbiome and the brain can occur across numerous physiological channels, including neuroendocrine and neuroimmune pathways and the autonomic nervous system (Cryan & Dinan, 2012; cf. Clarke, Stilling et al., 2014, for a review of endocrine effects of the gut microbiota), and bacteria found within the gut can produce neurotransmitters that can also be found within the central nervous system (Wall et al., 2014). For example, evidence suggests that a strain of *Lactobacillus brevis* can produce GABA (Barrett, Ross, O'Toole, Fitzgerald, & Stanton, 2012). Monoamines play a key role in brain–gut–microbiome axis signalling (Lyte, 2013); this includes serotonin (as well as its precursor tryptophan; O'Mahony, Clarke, Borre, Dinan, & Cryan, 2015), a key target in the treatment of major depression. Strikingly, initial evidence suggests that that the diversity of the gut microbiota may be related to brain structure as well (Fernandez‐Real et al., 2015). This bidirectional communication between the brain and the gut microbiota can impact upon stress (De Palma, Collins, Bercik, & Verdu, 2014), as well as cognition (Gareau, 2014); issues which we discuss further in the next two sections.

## 3 PSYCHOLOGICAL STRESS, HOST PHYSIOLOGY, AND THE GUT MICROBIOME: A RECIPROCAL RELATIONSHIP

Stress has been conceptualised as a challenge or threat which can disrupt the homeostasis of an organism (for recent discussion, see Romero, Dickens, & Cyr, 2009). Disruption to homeostatic physiological states is likely to include the ecosystem within the gastrointestinal tract. Stress can alter the composition and function of the gut microbiota (e.g., Bailey et al., 2011); there is even evidence from laboratory animal models that stress in mothers during prenatal development can impact upon the gut microbiota as well as other major physiological systems in their offspring (Golubeva et al., 2015). Further dysregulation can occur due to chronic stress in early life and into adulthood. Much emphasis has been placed on social/emotional contagion via parents in transmitting anxiety to children, such as the psychoanalytic work of Harry Stack Sullivan (1953), as well as theories emphasising modelling of behaviour and parental acceptance and control (cf. Wood, McLeod, Sigman, Hwang, & Chu, 2003). Nonetheless, a propensity to heightened anxiety may also be transmitted biologically, not only through human genes, but also via the microbiota via vaginal delivery (see section on psychological development below).

The microbiota can in turn impact upon the stress response. Germ‐free rodents lack a gut microbiota and are thus a useful means of examining the impact of the microbiota as a whole upon stress and cognition (Luczynski, Neufeld, et al., 2016 ). Germ‐free mice have shown altered anxiety‐like behaviour, suggesting a link between the microbiota and stress, although the evidence is equivocal with regard to whether it is increased or decreased (Clarke et al., 2013; Crumeyrolle‐Arias et al., 2014; De Palma et al., 2015). There is more consistent evidence of increased hypothalamic–pituitary–adrenal (HPA) axis activity in response to acute stress within these microbiota‐deficient animals (Luczynski, Neufeld, et al., 2016). Differences in stress within germ‐free rodents might be explained by changes in morphology of neuronal dendrites within the ventral hippocampus and basolateral amygdala (Luczynski, Whelan, et al., 2016).

The physiological impact of chronic stress can be contrasted with acute stress. For example, acute stress is associated with release of cortisol via the HPA axis (e.g., Allen, Kennedy, Cryan, Dinan, & Clarke, 2014; Allen, Kennedy, et al., 2017; Dickerson & Kemeny, 2004); an adaptive process that facilitates a response to immediate exposure to a stressor. In contrast, chronic stress is associated with dysregulation of the HPA axis (e.g., in people caring for family members with dementia, de Vugt et al., 2005; Stalder et al., 2014). Similarly, acute stress has been found to be associated with potentially adaptive immune responses (suppression of immunity mounted against intracellular pathogens, but preservation of immunity mounted against extracellular pathogens), whereas chronic stress has been associated with a maladaptive suppression of both types of immune response (Segerstrom & Miller, 2004). A key characteristic of dysfunctional stress is thus the inability to shut down an acute stress response; for example, in irritable bowel syndrome (IBS), a disorder of the brain–gut–microbiome axis, HPA axis activation persists for longer than in healthy controls (Kennedy, Cryan, Quigley, et al., 2014a; see section on IBS below). The impact of stress on the autonomic nervous system is evident in the association between high levels of stress at work and vagally‐mediated heart rate variability (Jarczok et al., 2013). The role of the vagus nerve is particularly interesting here, as evidence suggests that gut‐brain signalling may be mediated by the vagus nerve (Bravo et al., 2011).

Given this frequent dissociation between acute and chronic stress in how they impact upon stress biomarkers, it is of interest whether acute and chronic stress will have different effects upon the gut and its microbiota. However, it is likely that acute stress effects on the microbiota may be limited by the relative stability of the micobiota over time, though persistent stressors may disturb this “equilibrium” (Lozupone, Stombaugh, Gordon, Jansson, & Knight, 2012). It is perhaps not surprising that this question has not been well studied in humans, as researchers lack in vivo methods for studying the composition and function of the gut microbiota; assessment typically requires the collection of stool samples.

Given the strong evidence from laboratory animal models that stress impacts upon the brain–gut–microbiome axis, this area requires more research in humans. Such an endeavour will be guided by psychological models of stress that capture factors such as individual differences (e.g., Mark & Smith, 2008) as well as dynamic processes such as coping mechanisms employed in response to stress (e.g., Skinner, Edge, Altman, & Sherwood, 2003). Evidence from laboratory animal models has also provided a rationale for examining the role of the gut microbiota in stress‐related disorders in humans, both in terms of differences between clinical groups and healthy controls, as well as whether changes within the gut may mediate amelioration of psychological disorder (e.g., Nowakowski et al., 2016). For example, evidence indicates that the gut microbiota is altered in patients with major depression (Jiang et al., 2015; Kelly, Borre, et al., 2016; Naseribafrouei et al., 2014), as well as IBS (see section below). Despite these promising initial findings on the microbiota and stress‐related disorders, there has been a relative lack of research within healthy individuals linking the composition and function of the human gut microbiota to levels of chronic stress, or to susceptibility to acute stress. Such evidence, particularly if done in a longitudinal design, would have the potential to inform us on the mediating role of the gut microbiota in the progression from chronic stress to stress‐related disorders.

## 4 COGNITION AND THE GUT MICROBIOME

The gut microbiota has been shown to interact with host cognition in numerous laboratory animal model studies. Germ‐free animals have shown reduced anxiety‐like behaviour as well as changes in NMDA receptor subunits in the amygdala and increased hippocampal brain‐derived neurotrophic factor (a protein associated with neurogenesis; Neufeld, Kang, Bienenstock, & Foster, 2011) and antimicrobial administration increased hippocampal expression of brain‐derived neurotrophic factor (Bercik et al., 2011). Consistent with this impact upon the hippocampus, germ‐free rodents have been shown to have impaired short‐term recognition and working memory (Gareau et al., 2010). Germ‐fee animals have also displayed altered social behaviour (Arentsen, Raith, Qian, Forssberg, & Diaz‐Heijtz, 2015; Desbonnet, Clarke, Shanahan, Dinan, & Cryan, 2014), and intriguingly, Desbonnet et al. found that recolonisation restored social preference but not social cognition. Furthermore, maternal high‐fat diet had a negative impact on mice offspring's social behaviour that was reversed by treatment with *Lactobacillus reuteri* (Buffington et al., 2016). Such changes could be attributable to neurological changes such as increased myelination in the prefrontal cortex in germ‐free mice, a change which could be reversed by the restoration of the microbiota (Hoban et al., 2016).

Research in animal models will be crucial in guiding research in the human brain–gut–microbiome axis, as the impact of microbiota on specific brain regions and aspects of animal behaviour will help in the selection of cognitive tasks to explore. Research employing animal models will also be useful in identifying which bacteria may be of particular relevance. For example, in rodent models, a specific strain of *Bifidobacterium longum* was found to alter cognition (Savignac, Tramullas, Kiely, Dinan, & Cryan, 2015), as well as stress‐related behaviour and physiology (Savignac, Kiely, Dinan, & Cryan, 2014), and a similar profile of effects was subsequently observed in humans who were given this strain (Allen, Hutch, et al., 2016; see section below on psychobiotics). However, despite promising results from preclinical investigation (Bravo et al., 2011), such effects were not evident when healthy human volunteers consumed a strain of *Lactobacillus* (Kelly, Allen, et al., 2017); this suggests differences between bacterial strains in the degree to which they can be translated from laboratory animal models to humans.

Despite this potential for translational application of research findings from laboratory animal models to human psychology, there has been a relative lack of research linking the overall structure and composition of the microbiome to cognition in humans. Nonetheless, evidence that cognitive performance in humans can be moderated by probiotics (see section below) is further indicative of a role of the microbiota in cognition. As some of these effects may be relatively subtle, particularly in healthy young adults (Allen, Hutch, et al., 2016; Tillisch, 2014), there is clearly scope for cognitive psychologists to investigate further how specific aspects of cognition may be affected by the brain–gut–microbiome axis, using well‐specified measures of cognitive performance. For example, although some evidence suggests that probiotic intervention can alter sustained attention (Chung et al., 2014), overall sustained attention performance may not be as informative as time‐on‐task data that tracks sustained attention performance over time, when performance is likely to deteriorate (Allen & Smith, 2012; Mackworth, 1948; Verster & Roth, 2013).

Given the mounting preclinical evidence for its impact upon psychological function, the gut microbiota has been provocatively described as part of the unconscious system(s) influencing behaviour (Dinan, Stilling, Stanton, & Cryan, 2015), along with other physiological processes upon which a person may lack insight into, but nonetheless impact upon human psychology. We may further speculate that changes in the gut microbiota (e.g., due to antibiotic use), which are sufficient to alter consciously tangible factors such as bowel habit or gastrointestinal discomfort, may in turn alter interpretations of thoughts or emotions, consistent with the view that visceral factors impact upon human psychology (e.g. Loewenstein, 1996). The gut may thus have both conscious as well as unconscious effects upon psychological processes. These conscious effects are more likely to impact as an affect heuristic (Slovic, Finucane, Peters, & MacGregor, 2007); visceral factors act as a heuristic or mental shortcut (a Type 1 process), rather than as formal or logical information processing (Type 2 processes). They may nevertheless impact upon Type 2 processes; for example, by disrupting them. Models surrounding the interaction between Type 1 and Type 2 processes in thinking (e.g., Evans, 2007; Pennycook, Fugelsang, & Koehler, 2015) will be informative with regard to how the Type 1 factors stemming from the gut may impact upon Type 2 processing.

## 5 THE GUT MICROBIOTA AND PSYCHOLOGICAL DEVELOPMENT

There may be critical windows or sensitive periods for development of the gut microbiota (Borre et al., 2014), similar to the development of psychological functions such as language (e.g. Kuhl, Conboy, Padden, Nelson, & Pruitt, 2005; Werker & Tees, 2005). Human psychological and microbial development may be impacted by perinatal factors. The microbiota in rectal swabs of vaginally‐delivered infants differ from those delivered by caesarean section (Adlerberth et al., 2006), although infants born by caesarean section progressed to having a microbiota similar to their vaginally delivered counterparts by 8 weeks after birth (Hill et al., 2017). This is presumably due to a lack of microbial exposure in the womb; initial exposure to the vaginal microbiome on birth may lead to a substantially different population of microbes colonising the gut compared to C‐section delivery, which does not deliver this exposure. Such changes may have implication for public health, given the high prevalence of unnecessary caesarean sections being performed in many countries worldwide (Gibbons et al., 2010). Other factors shortly after birth could also affect the microbiota, such as the use of antibiotics in neonatal intensive care, and feeding pattern, which may in turn impact upon neurobiological development (cf. reviews by Adlerberth & Wold, 2009; Clarke, O'Mahony, Dinan, & Cryan, 2014).

### 5.1 The psychology of aging and the gut microbiome

Research indicates that the gut microbiota is associated with health in the elderly, with those in long‐term care having a less diverse microbiota than those living in the community (Claesson et al., 2012), although there is substantial distinctiveness at an interindividual level (Claesson et al., 2011). Even under conditions of healthy ageing, there can be a decline in some aspects of cognition (e.g., Koen & Yonelinas, 2014), but given the rising global health burden of dementia (Prince et al., 2015), as well as the psychological and physiological toll this can take upon caregivers (e.g., Allen, Curran et al., 2017; Etters, Goodall, & Harrison, 2008; Kiecolt‐Glaser, Dura, Speicher, Trask, & Glaser, 1991) a greater understanding of how gut health may impact upon the psychological health in ageing is critical. Despite this initial interest in gut microbiota changes with advancing age, there has been a relative lack of long‐term longitudinal research examining changes in the human gut microbiota. This is unfortunate given the high interindividual variation in the gut microbiota. Such research efforts would occur in the context of rapid acceleration of genetic sequencing technologies used to better characterise the gut microbiota (e.g., Mardis, 2008; Reuter, Spacek, & Snyder, 2015).

## 6 SOCIETY, CULTURE, AND THE MICROBIOME

“It is easy for an academic at a round table to claim that we live in a post‐ideological universe, but the moment he (sic) visits the lavatory after the heated discussion, he is again knee‐deep in ideology”

Slavoj Žižek (1997), *The Plague of Fantasies*

A “bottom‐up” approach to the brain–gut–microbiome axis will offer greater insight into the mechanisms whereby molecular factors drive interactions between the gut microbiome, brain function and human psychology. Although this will indicate directions for intervention within this axis, rapid changes in public health will also result from a macroscopic understanding of how broader trends at an interpersonal, societal, and cultural level impact upon the brain–gut–microbiome axis.

### 6.1 Social behaviour

Emerging research suggests that the gut microbiota impact upon social behaviour; evidence of altered social behaviour in germ‐free animals (Desbonnet et al., 2014) is complemented by evidence indicating that children with autistic spectrum disorder (ASD) have a gut microbial profile that differs from that of controls (Finegold et al., 2010; Wang et al., 2011). A double‐blind, placebo‐controlled trial of probiotic supplementation on children with ASD did not find an impact upon behavioural symptoms, although the study suffered from a low sample size (Parracho et al., 2010). It should be noted that this is an area prone to controversy, due not only to the question of the microbiota in ASD, but to the underlying debate around how ASD is defined as a disorder (e.g., Jaarsma & Welin, 2012). The immune pathway within the brain–gut–microbiome axis may be a plausible mediator of the effects of this axis on social behaviour, as cytokine‐induced sickness behaviour is associated with social withdrawal (e.g., Dantzer, O'Connor, Freund, Johnson, & Kelley, 2008). Conversely, social threat may lead to a proinflammatory immune response (Slavich & Irwin, 2014).

### 6.2 Culture

The brain‐gut‐microbiome axis may not only affect interindividual social psychology, but can also be placed in a broader social and cultural context. Globalisation may impact on the global homogeneity of diet (e.g., World Health Organization, 2002). A “Western” diet (high in carbohydrates and processed foods) may have a broad impact upon the gut microbiota. An increasing evidence base suggests differing (often more diverse) microbiota in populations with diets that differ substantially from Western populations, including hunter‐gatherers in the Chihuahuan Desert (Leach & Sobolik, 2010) the Hazda of Tanzania (Schnorr et al., 2014), the Asaro and Suasi people of Papua New Guinea (Martínez et al., 2015), and children from a rural village in Burkina Faso, where the diet is similar to when agriculture and animal husbandry were introduced c. 10,000 years ago (De Filippo et al., 2010). The increased international prevalence of the Western diet (e.g., Pingali, 2007) may have implications for public psychological health, and this may be mediated via the brain–gut–microbiome axis.

Despite this increasing dietary homogeneity, substantial changes in diet are still likely within individuals who travel frequently. Case evidence has demonstrated profound changes in the microbiota following intercontinental travel, which are reversible upon return (David et al., 2014). It is of interest whether such frequent, short‐term changes in the gut microbiota are associated with transient changes in psychological variables. Such questions obviously need to consider the large number of changes occurring during international travel. Nor should we ignore the impact of our physical environments, much of which is determined by urban and suburban planning; indoor environments are generally designed to reduce microbial diversity (Lax, Nagler, & Gilbert, 2015), yet research indicates that people with immune disorder (atopic individuals) have reduced exposure to environmental biodiversity and lower diversity in skin microbiota were observed (Hanski et al., 2012); the microbiome of the built environment may impact upon mental health (Hoisington, Brenner, Kinney, Postolache, & Lowry, 2015).

Notwithstanding the impact of globalisation, cultural factors continue to not only impact upon the interpretation and reporting of symptoms of psychological disorders (e.g., MacLachlan, 2006), but also of functional gastrointestinal disorders (cf. Francisconi et al., 2016). This is relevant for physicians working in increasingly multicultural contexts, as well as for international comparisons of disorders of the brain–gut–microbiome axis; researchers need to consider if disorders may be diagnosed with differing frequencies in different regions due to cultural factors.

**Figure 1:**
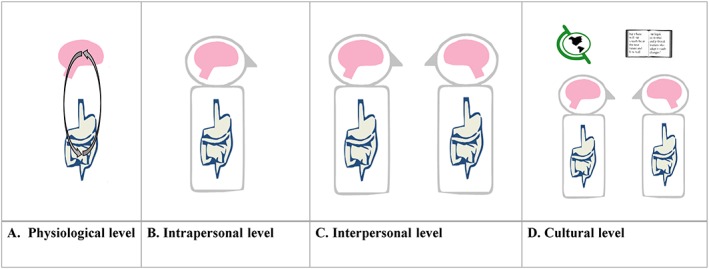
Psychology of the brain–gut–microbiome axis. (a) At a physiological level, the brain and gut microbiome interact through various physiological mechanisms, influencing psychological factors such as cognition and stress. (b) The individual interprets signals or symptoms arising from the physiological mechanisms of the BGM axis, for example, symptoms of irritable bowel syndrome, through cognitive schemas developed throughout life. (c). At the interindividual level, the BGM axis interacts with interpersonal psychological processes and the microbiome may be transferred from one individual to the next (see section on fecal microbiota transplantation)‐resultant change within the microbiota has the potential to impact upon psychological factors. (d). The workings of the BGM axis occur within a broader cultural context, and factors such as globalisation and societal change may moderate how the gut microbiota affect thought and behaviour, both under conditions of well‐being and in the context of psychological disorder.

In sum, a psychology of the brain–gut–microbiome axis should utilise multiple levels of analysis to understand this axis, not only at a physiological and intrapersonal level but also at a social and cultural level (See Figure 1).

## 7 CHANGING OUR MINDS, CHANGING OUR GUTS

“We should mind our microbes and they will mind us”

Fergus Shanahan, *The Unloved Gut* (2016, Hekoten International, 8[3])

### 7.1 Nutritional Psychology

As highlighted above, different diets will impact upon the composition of the gut. A vegan diet has been found to be associated with a reduction in potentially pathological microbiota (Glick‐Bauer & Yeh, 2014), while in contrast, a high‐fat diet may be associated with alterations in the microbiota that could lead to an increased risk of inflammation (Murphy, Velazquez, & Herbert, 2015). Furthermore, certain bacteria may flourish when certain nutrients are consumed in higher quantities; this is likely to have implications for the brain–gut–microbiome axis (Oriach, Robertson, Stanton, Cryan, & Dinan, 2016), and this may in turn impact upon health. Given the need for a diversity of microorganisms to utilise dietary macronutrients (e.g., Cantarel, Lombard, & Henrissat, 2012; Pokusaeva, Fitzgerald, & van Sinderen, 2011), diversity of microbiota is likely to mediate the impact of diet on host health (Conlon & Bird, 2015). Behavioural interventions targeted towards maintaining a healthy diet may thus lead to positive psychological effects that are mediated through gut microbiome effects. Indeed, the discipline of psychiatry is increasingly recognising that nutrition is a key factor in improving mental as well as physical health (Sandhu et al., 2017; Sarris et al., 2015).

In addition to broader dietary trends, there has been an increasing research interest in the role of polyunsaturated fatty acids; they have been shown in laboratory animal models to alter microbial composition as well as improving cognition and dampening HPA axis activity (Robertson et al., 2017; Yu et al., 2014) and to enhance adherence of probiotic bacteria to the gastrointestinal tract (Bomba et al., 2002). Research in humans indicates that docosahexaenoic acid, eicosapentaenoic acid, and total omega‐3 polyunsaturated fatty acids are lower in people with major depression compared to controls (Lin, Huang, & Su, 2010), and omega‐3 fatty acids have been shown to have a positive impact upon depression compared to placebo (Su, Huang, Chiu, & Shen, 2003). Further research is required on whether changes in the microbiota may mediate any impact of polyunsaturated fatty acids on psychological well‐being.

### 7.2 The impact of psychobiotics

A probiotic is a living microorganism that when ingested in sufficient quantities can ameliorate the health of the host; a psychobiotic was originally defined as a probiotic with a positive impact upon mental health (Dinan, Stanton, & Cryan, 2013) but has been broadened to encompass any microbiome‐mediated strategy that can modify mental health and psychological performance (Sarkar et al., 2016). Trials of putative psychobiotics have sought to demonstrate an impact of supplementation on stress, mood, and cognition. In healthy volunteers, probiotic consumption has been shown to improve mood, but only in those who have poorer mood at baseline (Benton, Williams, & Brown, 2007) and reduce depression and urinary free cortisol (Messaoudi et al., 2011). A single probiotic strain has been shown to reduce cortisol output in response to an acute stressor (Allen, Hutch, et al., 2016). A probiotic fermented milk drink has been shown to alter brain activity when processing information related to emotional facial expressions (Tillisch et al., 2013). Unfortunately, the latter study did not report on performance on the test, although another study indicated that fermented milk of a *Lactobaccillus helveticus* led to improvement in sustained attention in healthy older adults (Chung et al., 2014), and a recent study has indicated changes in frontal EEG mobility and associated changes in paired associate learning memory performance (Allen, Hutch, et al., 2016). It will be of interest for future research to examine potential mechanisms for these effects, although it should be noted that multiple mechanisms of probiotic bacteria are likely (e.g., Cryan & Dinan, 2012), including immune system effects, vagus nerve activation, tryptophan metabolism, and microbial metabolites. Probiotic bacteria can promote the production of neuroactive substances such as serotonin (Yano et al., 2015) and GABA (Barrett et al., 2012), which can impact upon psychological health, although these are unlikely to cross the blood–brain barrier and affect the brain directly.

Probiotics may be employed under conditions of disorder as well. In patients with IBS (see section below), probiotic intervention has been shown to ameliorate symptoms (e.g., Whorwell et al., 2006), including a reduction of abdominal pain in children (Giannetti et al., 2017). There is some evidence that probiotics can ameliorate anxiety and depressive symptoms (Pirbaglou et al., 2016), although research has been more likely to examine depressive symptoms in volunteers who do not necessarily suffer from clinical depression (Huang, Wang, & Hu, 2016). Recent research has examined the impact of probiotic intervention in major depression, finding that consumption of a probiotic cocktail was associated with reduced reported depression on the Beck Depression Inventory (Akkasheh et al., 2016).

Probiotic trials generally only administer a certain number of species in a certain concentration. Although a probiotic or antibiotic may impact upon the psychology of the host, it is difficult to tell how a probiotic is interacting with the other microbial species within the host, and although antibiotics may lead to more sweeping but transient effects on the human gut microbiota, they are not likely to ablate the microbiota completely. Caution is therefore necessary in drawing comparisons between extreme animal models in the laboratory, such as germ‐free rodents, and probiotic trials in humans.

### 7.3 Fecal microbiota transplantation

A more drastic means of altering the microbiota than administering a probiotic is transplanting the fecal microbiota of a donor. Such a method has shown success in treating patients with *Clostridium difficile* infection (Kassam, Lee, Yuan, & Hunt, 2013). If such a method can be used to produce desirable changes in the microbiota, one may speculate that it could do the same for the brain–gut–microbiome axis. Indeed, evidence indicates that symptoms of IBS may be ameliorated by fecal microbiota transplantation (cf. review by Smits, Bouter, de Vos, Borody, & Nieuwdorp, 2013). Germ‐free or antibiotic‐treated rodents can be colonised with stool from human conditions. For example, rodents colonised with microbiota from people suffering from depression and healthy controls exhibited differences in depression‐like and anxiety‐like behaviours (Kelly, Borre, et al., 2016; Zheng et al., 2016). By directly manipulating the microbiota in this manner, this approach allows for a more causal and mechanistic examination of the impact of the microbiota than would be possible in a human trial.

### 7.4 Psychological therapies

Conversely, we may pose the question of whether psychological or behavioural interventions may impact upon gut physiology and the gut microbiota, a key role for top‐down processes regulating the gut microbiota in the brain–gut–microbiome axis. Although the symptoms of IBS are gastrointestinal (see section on IBS below), cognitive behavioural therapy as well as hypnotherapy (Miller et al., 2015) have been successfully employed as an intervention for reducing IBS symptoms. There is currently limited evidence that psychological interventions may impact upon the composition of the gut microbiota to “normalise” it. However, mindfulness‐based stress reduction represents a promising psychological intervention for tackling chronic stress, for example in carers for persons with dementia (Whitebird et al., 2013). Comprehensive interventions that simultaneously target diet and other lifestyle factors such as exercise, in addition to cognition, behaviour, and psychological well‐being are likely to have the clearest impact upon the gut microbiota, although the specific effects of each aspect of treatment are challenging to untangle. Behavioural interventions that target diet in particular are likely to have major inroads into ameliorating dysfunction within the brain–gut–microbiome axis.

## 8 THE PSYCHOLOGY OF IRRITABLE BOWEL SYNDROME AND THE GUT MICROBIOME

“I was in pain for so long that I didn't care if I was in a band, I didn't care if I was alive”

Kurt Cobain (Nirvana), on suffering from irritable bowel syndrome

IBS is a functional gastrointestinal disorder associated with abdominal pain and altered bowel habits. Functional gastrointestinal disorders in general can be understood from a biopsychosocial perspective (Van Oudenhove et al., 2016), and this is particularly true of IBS: there is a high prevalence of early life stress in IBS patients compared to inflammatory bowel disease, an organic disease with comparable symptoms to IBS (Jemelka & Russo, 1993; Reilly, Baker, Rhodes, & Salmon, 1999). IBS is also associated with a high rate of comorbid anxiety and depression (Whitehead, Palsson, & Jones, 2002), and IBS is more prevalent among people exposed to the chronic stressor of caregiving for patients with serious illness (Remes‐Troche, Torres‐Aguilera, Montes‐Martínez, Jiménez‐García, & Roesch‐Dietlen, 2015). Research has also found that IBS is associated with altered cognition (Kennedy, Clarke, et al., 2014b; Kennedy et al., 2015) as well as an altered acute stress response (Kennedy, Cryan, Quigley, et al., 2014a; Suárez‐Hitz et al., 2012). It can thus be considered as a stress‐related brain–gut–microbiome disorder (Eisenstein, 2016; Grayson, 2016; Kennedy, Cryan, Dinan, et al., 2014c). As a condition that can be associated with heightened visceral sensitivity (Dong et al., 2004; Zhou & Verne, 2011), it may help us to better understand the embodiment of disorder.

IBS is associated with an altered microbial profile, but interestingly, those with IBS and a comorbid psychiatric disorder were more similar to healthy controls than they were to IBS patients without a comorbid psychiatry disorder (Jeffery et al., 2012). It thus seems that the symptom profile of IBS may stem primarily either from a disruption at a psychological level or at the microbial or gastrointestinal level. This microbial data chimes with prospective research, which observed both that mood disorders are predictive of developing functional gastrointestinal disorder, and that functional gastrointestinal disorder is predictive of developing mood disorders (Koloski, Jones, & Talley, 2016).

The cognitive–behavioural model of IBS suggests that cognitions relating to both gastrointestinal symptoms and psychological events are interpreted in a manner that may exacerbate their impact (Toner et al., 1998). IBS is associated with changes in psychological processes such as catastrophizing and hypervigilance to negative stimuli that may lead to maintenance of the disorder (see review by Kennedy et al., 2012). Consistent with this model of IBS, cognitive behavioural therapy has been shown to alleviate IBS symptoms and improve quality of life (Li, Xiong, Zhang, Yu, & Chen, 2014). It is likely that a greater understanding of the psychological processes underlying IBS will help in treatment of this psychological and gastrointestinal condition.

## 9 IMPLICATIONS FOR RESEARCH AND CLINICAL PSYCHOLOGY

Progress in the area of the brain–gut–microbiota axis requires multidisciplinary work that can elaborate on host–microbe interactions, further developing our knowledge of how this axis affects emotion and information processing, as well as how it interacts with the broader cultural context. See Table 1 for a description of key questions in this area.

**Table 1:** Key questions for a psychology of the brain–gut–microbiome axis

	Key questions
Cognitive psychology	How does the composition and function of the microbiota impact upon cognitive performance? Can the neurotransmitters produced by the gut microbiota impact upon stress and cognitive performance, and through what mechanism, if they cannot cross the blood‐brain barrier? How do visceral factors associated with the gastrointestinal tract impact upon cognitive function?
Social and cultural psychology	How does the composition and function of the microbiota impact upon social behaviour? Does social interaction impact upon the microbiota? How does culture interact with the presentation and treatment of disorders of the brain–gut–microbiota axis?
Clinical psychology	How is the composition and function of the microbiota altered under conditions of psychological disorder? Can interventions designed to target psychological well‐being alter the microbiota? Can interventions that ameliorate dysregulation of the microbiota improve psychological well‐being? How do functional gastrointestinal disorders interact with cognition, emotion, and stress?

There is evidence from large samples that the gut microbiota differs substantially between individuals, and that numerous behavioural factors are associated with the composition of the microbiota, although any one factor will only explain a small amount of the variance in the microbiota (Falony et al., 2016; Zhernakova et al., 2016). Given these cross‐sectional findings, future research is likely to have greater explanatory power if it focuses on changes within individuals (who are likely to exhibit less substantial variability in this microbial profile over time). More long‐term follow‐up of individuals across major transitions in their lifespan will allow for a better understanding of whether disruption of the microbiome during sensitive periods for microbiome development has implications for psychological development.

In a context of increased global mobility, longitudinal research may have a particular relevance for straddling the impact of social/environmental factors and physiological variables. People who move to different continents, particularly during early childhood or as elderly people, may be exposed to substantial changes in diet and environmental diversity that pose a particular challenge or opportunity for well‐being within the brain–gut–microbiome axis. Such a research endeavour would clearly involve substantial international collaboration.

Laboratory animal models are a key tool in studying causal mechanisms within the brain–gut–microbiome axis, as well as allowing for researchers to look at physiological processes in greater depth than would be possible within humans. However, laboratory animals generally live under abnormally hygienic conditions that are not representative of animals living in conditions such as in the wild, or in pet shops, with associated implications for immune system function, although cohousing laboratory animals with pet shop animals may reverse these effects (Beura et al., 2016). Research on animals in the field may provide more ecologically valid information on how animal behaviour impacts upon the microbiota and vice versa. For example, research on chimpanzees has indicated that social interactions affect the gut microbiota (Moeller et al., 2016). Nor should the study of animals in general be solely concerned with modelling human psychology. It is important to view psychology as a discipline that also studies the adaptive behaviour and cognition of other species as a subject in its own right, not only as a means for understanding mechanisms for human psychobiology (Staddon, 1989).

Within clinical practice, psychologists should be aware of the high comorbidity of psychological distress and gastrointestinal problems. There is likely much potential for the amelioration of gastrointestinal problems (e.g., through intervention in diet, probiotic supplementation or medical treatment) to assist in the management of psychological disorder and vice versa. Notwithstanding differences in the extent to which individuals seek psychological help in a manner consistent with their culture, greater awareness of cross‐cultural differences in reporting of gastrointestinal symptoms and psychological problems will assist in better‐targeted interventions for brain–gut–microbiome axis disorders.

If acknowledgement of the role of the brain–gut–microbiome axis can disrupt biases such as anthropocentrism and disembodiment within some branches of psychology (see section below), this will have implications not just for research and clinical practice, but also for the teaching of psychology and cognate disciplines such as neuroscience. Curricula will thus faces new challenges, and one of the implications of microbiome research is that such programs may need to place an increased emphasis on multidisciplinary approaches (e.g., a renewed focus on systems biology) and breaking down barriers between heretofore siloed disciplines.

## 10 THE MICROBIOME AND BEHAVIOUR: CHALLENGING PSYCHOLOGICAL CONSTRUCTS

Another clear objective of this review is to reframe the narrative within psychology as a discipline to challenge three biases:

- With ongoing improvements in neuroscientific technologies, increasing focus has been placed on the localisation of psychological function within specific brain regions and across brain circuits. Although this has led to great research productivity (e.g., Cabeza & Nyberg, 2000), it has not been without setbacks in technical realisation and statistical interpretation (e.g., Eklund, Nichols, & Knutsson, 2016; Vul, Harris, Winkielman & Pashler, 2009). Furthermore, such reductionistic approaches have been criticised at more conceptual levels, such as the need to consider the role of behaviour (e.g., Uttal, 2001) as well as the body (e.g., Kiverstein & Miller, 2015) in general. Moreover, the role of interoception and other parts of the human body with regard to psychology cannot be ignored (Craig, 2002).
- Recent trends in the study of human psychology have increasingly challenged a “disembodied” psychology, through an increased emphasis on the role of the human body and related processes in human psychology. For example, embodied cognition can be influenced by social processes such as language that impact upon the implicit understanding of the body and its boundaries (Borghi & Cimatti, 2010). The challenge of an embodied psychology must also be met at a methodological level, taking account of factors such as the physical, task‐relevant resources an agent possesses (Wilson & Golonka, 2013); microbial agents may provide a physical resource that can aid in adaptive behaviour and cognition. Artificial intelligence has been productive within cognitive science, and in the case of robotics, can be used to study cognition that is in some sense embodied and extended into the environment. Nonetheless, a key distinction between artificial intelligence and human psychology is that human psychology occurs within a visceral context. Visceral factors (e.g., hunger and sex drive) may cause humans to behave in a manner that goes against decision‐making based upon logical analysis of their situation (e.g., Loewenstein, 1996). As the goal‐directed behaviour of engineered artificial intelligence systems can be explained without reference to any microbes they may physically contain, such systems are unlikely to explain the psychology of organisms such as humans which have coevolved with microbes, and for whom there are plausible mechanisms for brain–microbe interactions.
- A related and longstanding concern within the discipline of psychology has been the risk of anthropocentrism, an excessive focus on humans, as well as anthropomorphism, a tendency to attribute human psychological traits to other animals with insufficient evidence for doing so (e.g., Barrett, 2015; Staddon, 1989; Yerkes, 1933). Host–microbe interactions in eukaryotic life extend back to before the emergence of our human species; for example, work in paleovirology has outlined a role for viruses in the evolution of placental mammals and other life forms (cf. Haig, 2015; Lavialle et al., 2013; Villarreal, 2011) and touches all aspects of the natural world (see Alivisatos et al., 2015; Yong, 2016). Given our shared evolutionary history with microbes (Stilling, Bordenstein, Dinan, & Cryan, 2014), it is to be expected that changes in human behaviours (e.g., eating behaviour, exercise, and sleep) and responses to psychological stressors, as well as mood and cognition, will be associated with changes in the gut microbiota, and vice versa (Alcock, Maley, & Aktipis, 2014; Stilling, Dinan, & Cryan, 2016).
  Within the study of our own species, the genetic information gleaned from the human genome project in mapping out the human genome (Lander et al., 2001) has had relatively limited success in explaining human psychology. This may lead to an underplaying of the role of genetics in human cognition, emotion, and behaviour (and, by extension, the role of the body and relevance of studying other species). It should, however, be noted that the majority of genes in the human body are not the genes of human cells, but rather are the genes of the microscopic organisms that dwell within humans—it has been estimated that the genes within the gut microbiota (i.e., the microbiome) alone comprise over 100 times more genes than those of human cells (e.g., Qin et al., 2010). Indeed, there has been some debate over whether humans (and other animals) can be thought of not as autonomous agents but as biomolecular networks or “holobionts,” with the host and microbial genomes of the holobiont collectively referred to as the “hologenome” (Bordenstein & Theis, 2015; Douglas & Werren, 2016, Theis et al., 2016).
  There is some evidence of horizontal gene transfer between the bacterial and human genome, suggesting a mechanism for close integration of the human and microbial genetics, although the number of bacterial genes for which this is relevant may be quite limited (Andersson, Doolittle, & Nesbø, 2001; Salzberg, White, Peterson, & Eisen, 2001). Compared to bacteria, genetic recombination between the viruses and host may be more relevant; up to 8% of the human genome is made up of human endogenous retrovirus genes (Griffiths, 2001), which may be associated with diseases of the central nervous system (Mortelmans, Wang‐Johanning, & Johanning, 2016), although this needs to be placed in a current context where very little is known about the human virome (Wylie, Weinstock, & Storch, 2012). Given the ability of microbes to engage in mechanisms that may affect psychological processes, such as the production of neuroactive metabolites and interaction with the host immune system, the impact of genetic factors on human psychology likely also extends to microbial genes. Furthermore, this microbial genetic information is relevant for identifying targets for clinical intervention, given that the microbial gene pool is more readily modifiable than the human component.

## 11 CONCLUSIONS

The human brain has long been described as one of the most complex structures in the known universe; sequencing the genome of the gut microbiota has revealed that the ecosystem of the gastrointestinal tract may be a close rival. Understanding how the brain and the gut microbiota interact at a functional level is likely to remain one of the great scientific challenges of the 21st century. The implications of this new endeavour need to be considered in the discipline of psychology as these findings are relevant across multiple areas. In parallel, a psychological perspective will help to take this research endeavour into new and exciting areas, and to apply the relevant findings in a more targeted manner, in clinical and research practice as well as in more general applications.
